# Artificial intelligence in liver cancer research: a scientometrics analysis of trends and topics

**DOI:** 10.3389/fonc.2024.1355454

**Published:** 2024-02-28

**Authors:** Mohammad Saeid Rezaee-Zavareh, Naomy Kim, Yee Hui Yeo, Hyunseok Kim, Jeong Min Lee, Claude B. Sirlin, Bachir Taouli, Rola Saouaf, Ashley M. Wachsman, Mazen Noureddin, Zhiping Wang, Jason Moore, Debiao Li, Amit G. Singal, Ju Dong Yang

**Affiliations:** ^1^ Middle East Liver Diseases Center, Tehran, Iran; ^2^ Karsh Division of Gastroenterology and Hepatology, Cedars-Sinai Medical Center, Los Angeles, CA, United States; ^3^ Comprehensive Transplant Center, Cedars-Sinai Medical Center, Los Angeles, CA, United States; ^4^ Department of Radiology, Seoul National University Hospital, Seoul, Republic of Korea; ^5^ Liver Imaging Group, Department of Radiology, University of California, San Diego, San Diego, CA, United States; ^6^ BioMedical Engineering and Imaging Institute (BMEII), Icahn School of Medicine at Mount Sinai, New York, NY, United States; ^7^ Department of Diagnostic, Molecular and Interventional Radiology, Icahn School of Medicine at Mount Sinai, New York, NY, United States; ^8^ Department of Radiology, Cedars-Sinai Medical Center, Los Angeles, CA, United States; ^9^ Department of Computational Biomedicine, Cedars-Sinai Medical Center, Los Angeles, CA, United States; ^10^ Department of Biomedical Sciences, Biomedical Imaging Research Institute, Cedars-Sinai Medical Center, Los Angeles, CA, United States; ^11^ Department of Internal Medicine, University of Texas Southwestern Medical Center, Dallas, TX, United States; ^12^ Samuel Oschin Comprehensive Cancer Institute, Cedars-Sinai Medical Center, Los Angeles, CA, United States

**Keywords:** liver cancer, artificial intelligence, machine learning, scientometrics, publication trend analysis

## Abstract

**Background and aims:**

With the rapid growth of artificial intelligence (AI) applications in various fields, understanding its impact on liver cancer research is paramount. This scientometrics project aims to investigate publication trends and topics in AI-related publications in liver cancer.

**Materials and Methods:**

We employed a search strategy to identify AI-related publications in liver cancer using Scopus database. We analyzed the number of publications, author affiliations, and journals that publish AI-related publications in liver cancer. Finally, the publications were grouped based on intended application.

**Results:**

We identified 3950 eligible publications (2695 articles, 366 reviews, and 889 other document types) from 1968 to August 3, 2023. There was a 12.7-fold increase in AI-related publications from 2013 to 2022. By comparison, the number of total publications on liver cancer increased by 1.7-fold. Our analysis revealed a significant shift in trends of AI-related publications on liver cancer in 2019. We also found a statistically significant consistent increase in numbers of AI-related publications over time (tau = 0.756, p < 0.0001). Eight (53%) of the top 15 journals with the most publications were radiology journals. The largest number of publications were from China (n=1156), the US (n=719), and Germany (n=236). The three most common publication categories were “medical image analysis for diagnosis” (37%), “diagnostic or prognostic biomarkers modeling & bioinformatics” (19%), and “genomic or molecular analysis” (18%).

**Conclusion:**

Our study reveals increasing interest in AI for liver cancer research, evidenced by a 12.7-fold growth in related publications over the past decade. A common application of AI is in medical imaging analysis for various purposes. China, the US, and Germany are leading contributors.

## Introduction

Artificial intelligence (AI) is a branch of computer science aiming to digitize and automate human activities (1). Since the first proposal to use AI in medicine in 1950s (2), it has been utilized in diverse domains of medicine (3). With the ongoing and gradually increasing influx of data across different medical domains, AI is expected to alter the landscape of medicine in various facets of clinical care, from patient diagnosis to treatment (3), and biomedical research.

Liver cancer is the third-leading cause of cancer-related deaths globally (4). A previous forecast analysis projected a rise of around 55% in liver cancer cases globally from 2020 to 2040 (5). To reduce the burden, innovative solutions are needed. Such solutions will need to span multiple domains, from liver cancer prevention, to early detection, noninvasive diagnosis and prognosis, and effective treatment and treatment response assessment. Due to the biological, epidemiological, and clinical heterogeneity of liver cancer, individualized approaches will be needed. In this context, AI can offer transformative solutions, from early detection to personalized treatment strategies (6).

The growth and advancement of a research field can be assessed by analyzing the rise in the number of related publications. Scientometrics is an approach that helps measure such growth or advancement. It involves a quantitative analysis of bibliometric metadata in scientific research (7). Previous scientometrics studies have analyzed the growth of AI in different fields of medicine (8–11) and in biomedicine as general (12). Recent scientometrics studies reviewed the impact of AI on liver cancer especially about the medical image diagnosis of cholangiocarcinoma (13, 14). However, no study based on the Scopus database, has evaluated AI in the entire continuum of liver cancer care from diagnosis to treatment, and for all types of liver cancer, including hepatocellular carcinoma (HCC) to determine the most active authors, institutions, journals, and countries in this field.

Determining active research networks on AI and liver cancer may lead to constructive collaborations and enable interested researchers from around the world to actively participate in these networks. Furthermore, by knowing these networks, companies in the medical industry may be more inclined to invest in them. Finally, understanding the current hot topics in the field of AI and liver cancer may help guide and direct future related research projects. Our primary aim is to utilize scientometrics to map publication trends concerning AI in liver cancer research. We aimed to report the areas of liver cancer that are most actively involved in AI research. We also sought to determine the most active countries and journals in this field.

## Materials and methods

### Search strategy and resources

We developed a search strategy to identify all liver cancer publications and all AI-related publications in the field of liver cancer on Scopus, one of the largest abstract and citation indexing databases (Appendix 1). To achieve this, we included all publications containing “liver cancer” and “artificial intelligence,” or similar terms, within their title, abstract, or keywords.

After running the search protocol in the Scopus database, we checked the accuracy of our search strategy by Google Scholar. Using a similar combination of the used keywords in our search protocol, we searched Google Scholar up to the first 20 pages and compared it with Scopus search results to ensure the completeness of our search strategy. Our last search for identifying related publications was performed on 3rd August 2023, without language limitation.

### Document types classification in the scopus database

We included all types of publications to assess the temporal trend in the number of AI research in liver cancer. Except for publication trends, we excluded publication types such as notes, letters, editorials, retracted publications, conference papers and reviews, books, and book chapters from the analysis.

### Determining authors affiliations

To determine the affiliations of the authors in the field of AI and liver cancer, we searched various research profiles of authors including Google Scholar and ORCID and the most recent publications of authors. We used the primary and most recent affiliation of the authors.

When a publication has authors from more than one country, it is considered an international collaboration. For example, if a publication has authors from country X and country Y, it will count for both countries.

### Top ten percent of publications by citation

First, we considered the total number of articles and reviews for our analysis to determine the most active authors, institutes, journals, and countries in the field of AI and liver cancer. We also checked 2022 journal citation report for determining impact factors (IF) of the journals. Next, we focused on the top 10% of publications by citation. To achieve this, we initially calculated a number equivalent to 10% of the total identified publications (articles and reviews) and then ranked them based on their citations in Scopus, resulting in the top 10% of publications by citation. Our last search for citation counting and analysis was performed on September 21^st^ 2023.

### Categorization of publication subjects

All identified publications, including only articles and reviews, were imported into EndNote software (version 21, Clarivate Analytics). We initially screened 50 to determine appropriate categories for publication classification. Subsequently, two authors (MSR-Z and NK) screened all publications to classify articles into the selected categories. Most categorization was based on title and abstract assessment, with full-text evaluation only utilized when title and abstract evaluation couldn’t precisely determine the most suitable category. Each publication was allowed to fall into more than one category. Any inconsistency was resolved through consultation with a third author (YHY or JDY). The final classification of publications was based on the following nine categories:

1- Diagnostic or Prognostic Biomarkers Modeling & Bioinformatics: Utilizing AI to analyze biological data, including single or multiple serum markers, possibly in combination with clinical and imaging information, to facilitate diagnosis or prognosis.
2- Drug Discovery or Drug-Related Model: Utilizing AI for drug discovery, drug development, identification of drug mechanisms, and the exploration of new treatment targets.
3- Genomic or Molecular Analysis: Utilizing AI to develop models for clinical applications, coupled with only molecular or genomic level analyses.
4- Histopathologic Diagnosis or Classification: Utilizing AI for histopathological diagnosis and classification.
5- Medical Image Analysis for Biopsy, Treatment Guiding or Planning: Utilizing AI to analyze and interpret medical images to guide and plan biopsies or treatments.
6- Medical Image Analysis for Diagnosis: Utilizing AI to analyze and interpret medical images for diagnosis or lesion detection.
7- Medical Image Analysis for Segmentation: Utilizing AI to outline and separate different parts of the image, like liver and other organ, liver anatomic segments, liver tumors, or blood vessels.
8- Radiomics: Utilizing AI to extract features from images offering insights into disease diagnosis, prognosis, and treatment.
9- Treatment Response, Survival or Recurrence Evaluation or Prediction: Utilizing AI to incorporate various inputs from clinical and practical data to assess or predict treatment outcomes, recurrence, mortality or survival.

### Statistical analysis

Descriptive analysis was conducted using Scopus analysis tool and Microsoft Excel 2021 to determine the most active authors, institutes, journals, and countries in publishing scientific documents in the field of AI and liver cancer. We determined the publications growth in the last decade (2013-2022) and also conducted Change-Point Analysis using R version 4.1.2 and the ‘changepoint’ package to identify any significant shifts in publication trends over different years. Additionally, we performed the Mann-Kendall Test to simply assess the correlation between AI-related publications on liver cancer and publication year. Finally, we calculated the percentage of AI-related publications on liver cancer as a portion of total liver cancer publications from 2000 to 2022. A significance level of P-value less than 0.05 was employed.

## Results

### Number of AI-related publications on liver cancer

**Figure 1:**
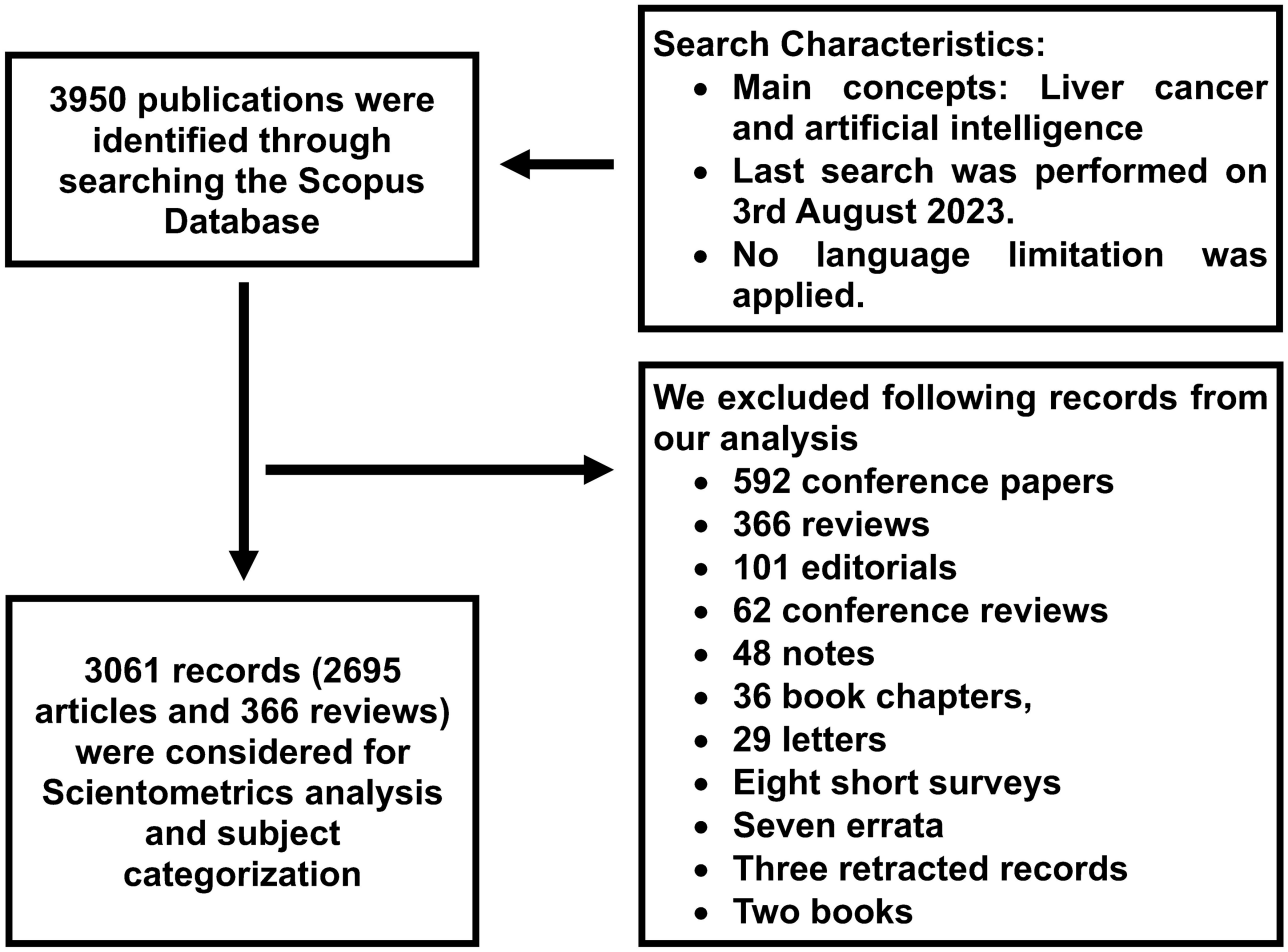
Flow Diagram of Study Identification.

Our search strategy identified 3950 publications including 2695 articles, 592 conference papers, 366 reviews, 101 editorials, 62 conference reviews, 48 notes, 36 book chapters, 29 letters, eight short surveys, seven errata, three retracted, two books, and one data paper (**Figure 1** and **Supplementary Figure 1**).

**Figure 2:**
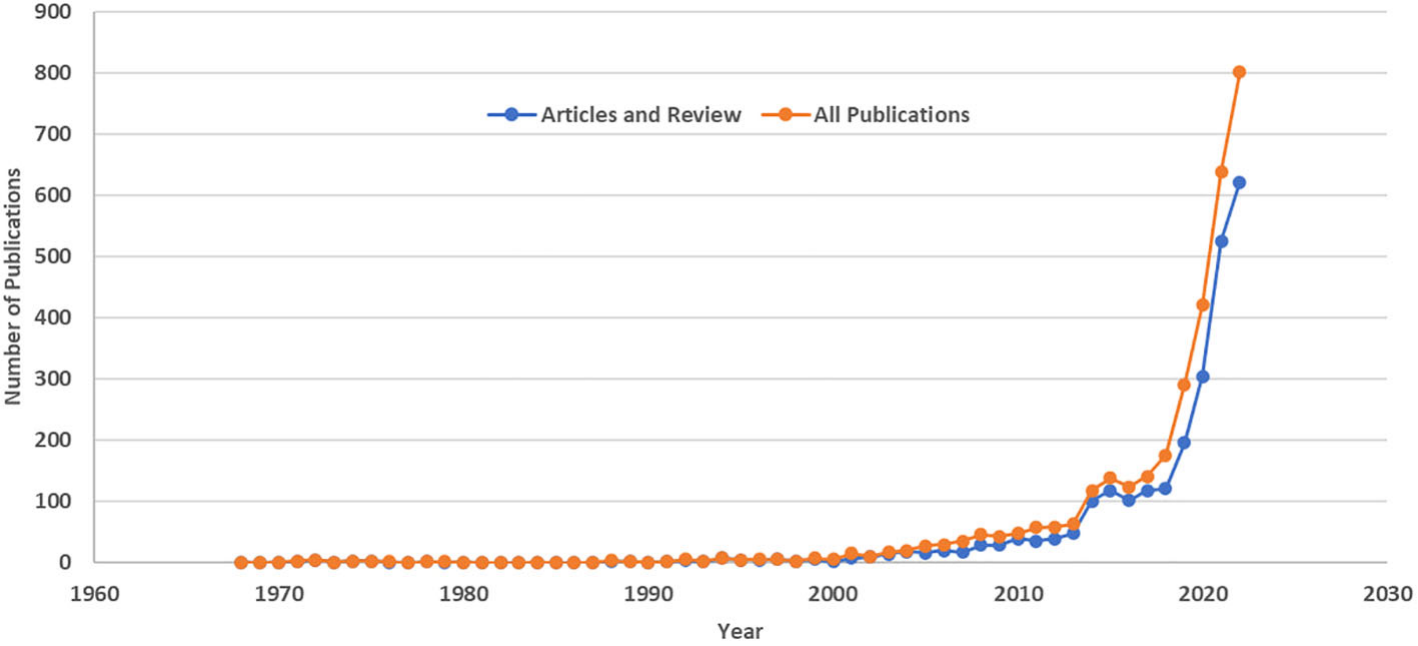
Artificial Intelligence-Related Publications on Liver Cancer Based on the Scopus Database: Publications by Year (1968-2022).

Based on our search strategy, the oldest publication on AI and liver cancer was published in 1968 (15). Between 1968 and 1999 (32 years), a total of 78 document were published in this field, which increased to 413 and 2913 between 2000-2012 (13 years) and 2013-2022 (10 years), respectively. Over the past decade, yearly publications increased from 63 in 2013 to 802 in 2022, indicating a 12.7-fold increase. This translates to, on average, one AI-related document in the field of liver cancer being published every 1.25 days (**Figure 2**).

**Figure 3:**
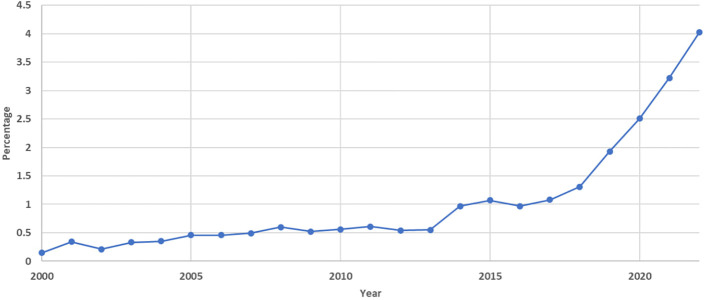
Annual Percentage of Artificial Intelligence-Related Publications on Liver Cancer as a Proportion of Total Liver Cancer Publications Based on the Scopus Database (2000-2022).

We also searched the Scopus database for liver cancer publications. There were 317,433 publications in total by the end of 2022. In 2013, there were 11,404, which increased to 19,889 in 2022, showing a 1.74-fold growth over the decade. This made AI-related publications go from 0.55% to 4.03% of all liver cancer publications during the past decade (**Figure 3**). A change-point analysis pinpointed a significant shift in publication trends, revealing a change in the mean of publication numbers in the year 2019. Additionally, the Mann-Kendall test identified a statistically significant positive trend in publication numbers, indicating a consistent increase over time (tau = 0.756, p < 0.0001).

When considering only the top 10% of publications by citation, we found that 210 out of 306 categorized in the top 10 citations were published during 2013-2022. Additionally, 86 other publications in this category were published between 2003 and 2012, and only 10 publications were published before 2003. In addition to the number of publications in the top 10% most cited, we evaluated the percentage of published papers in this category for each year relative to the whole number of publications in that year. We observed a decreasing trend in the percentage of these publications starting from 2003. (**Supplementary Figure 2**).

### Journals, author, institutions, and countries with the largest number of AI publications in liver cancer

**Figure 4:**
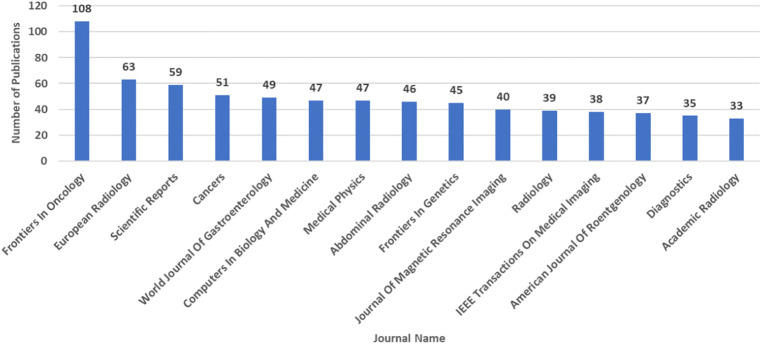
Number of Artificial Intelligence-Related Publications (Articles and Reviews) on Liver Cancer Based on the Scopus Database by 3^rd^ August 2023: Top 15 Journals.

**Figure 5:**
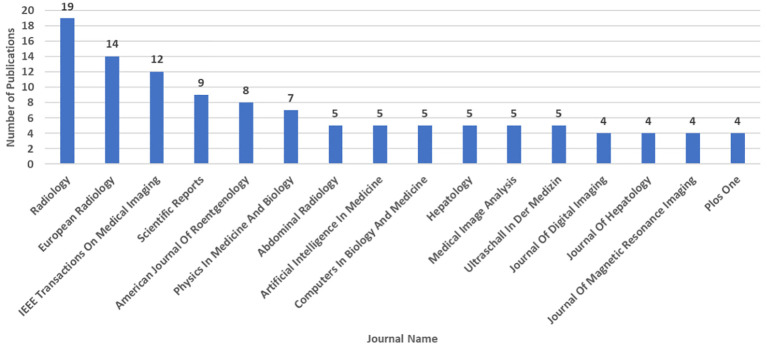
Number of Top 10% of Artificial Intelligence-Related Publications (Articles and Reviews) on Liver Cancer by Citation Based on the Scopus Database by September 21^st^ 2023: Top 16 Journals (Tied for 15^th^ Place).

For determining journals (and all other analyses hereafter) with the largest number of publications in the field of AI and liver cancer, we only considered articles and reviews and excluded other types of publications from our investigation. **Figure 4** shows the top 15 journals with the most publications of AI-related documents on liver cancer. Eight (53%) are radiology journals, with minimum, mean, and maximum 2022 IFs of 2.4, 6.0, and 19.7, respectively. In **Figure 5**, which focuses solely on the top 10% of publications by citation, the journal ‘Radiology’ claimed the top position.

The top 15 prolific authors in the field of AI and liver cancer (**Supplementary Table 1**). were from the US (n=5), China (n=4), South Korea (n=4), France (n=1), and Switzerland (n=1). The number of AI-related articles and reviews on liver cancer, total publications, and H-index of these authors (at the time of data extraction from Scopus) are shown in **Supplementary Table 1**. The Chinese Academy of Sciences (n=85), Ministry of Education China (n=84), and Fudan University (n=84) have had the most activity in publishing AI-related articles and reviews on liver cancer. The top 10 institutions with the greatest number of publications in this field are from France (n=1), the US (n=1), and China (n=8) (**Supplementary Figure 3**).

**Figure 6:**
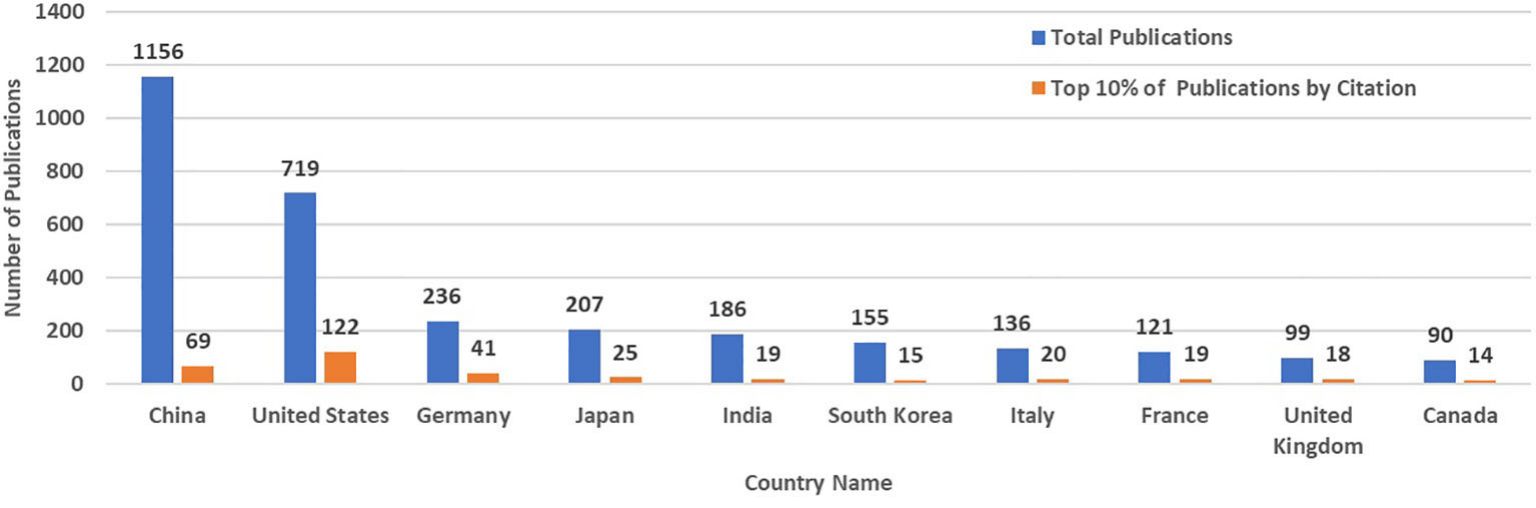
Comparison of Artificial Intelligence-Related Publications on Liver Cancer: Total vs. Top 10% by Citation in Top 10 Countries Based on the Scopus Database by September 21^st^ 2023.

China had 1156 articles and reviews in this field. US (n=719), Germany (n=236), Japan (n=207), India (n=186), South Korea (n=155), Italy (n=136), France (n=121), UK (n=99), and Canada (n=90) are the next nine countries with the largest number of publications. However, when considering only the top 10% of publications by citation, the US emerged as a major contributor, with 122 out of the 306 publications (39.9%). The rankings for other countries changed, with China (69), Germany (41), Japan (25), Italy (20), France (19), India (19), the United Kingdom (18), and Canada (14). (**Figure 6**).

### Categorization of publications

**Figure 7:**
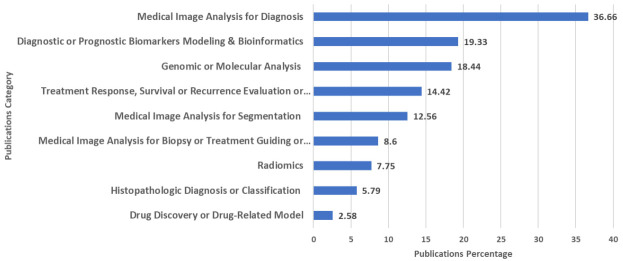
Percentage of Artificial Intelligence-Related Publications (Articles and Reviews) on Liver Cancer Across Various Categories Based on the Scopus Database by 3^rd^ August 2023.

The majority of publications identified within this project were linked to medical image analysis (**Figure 7**). The category that demonstrated the highest percentage of evaluated publications was “medical image analysis for diagnosis” (37%). Additionally, 13% and 9% of publications were associated with image analysis for segmentation or for guiding or planning biopsies and therapies, respectively. The three next most frequent categories were “diagnostic or prognostic biomarkers modeling & bioinformatics” (19%), “genomics or molecular analysis” (18%), and “treatment response, survival, or recurrence evaluation or prediction” (14%).

## Discussion

We performed a scientometrics analysis of all AI-related publications in liver cancer to ascertain the authors, institutions, journals, and countries with the highest level of activity in this field. Our investigation revealed a substantial growth in the number of these publications, increasing by 12.7-fold in the past decade from 2013 to 2022. Our findings underscore the dynamic nature of research activity in the field of AI and liver cancer, with a notable change in 2019 and a sustained upward trajectory in publication output. Although the number of top 10% most cited publications on AI and liver cancer shows an increasing trend, we noted a decreasing trend in the percentage of these publications. While newer publications may not have had sufficient time to accumulate citations, the decreasing trend in the percentage of these publications is evident from 2003 to 2022. While evaluating other citation percentage categories like top 20 or top 50% seems reasonable for future research project, our analysis suggests that our resources, including time and funding, should be prioritized towards performing high-quality research project.

While recent scientometrics studies, based on Web of Science (14, 16), highlighted the rising trend of AI publications in the field of liver cancer, our search strategy proved to be more sensitive. It allowed us to identify a greater number of articles and reviews from an earlier time period, starting from 1968.

As we observed in the liver cancer field, a considerable growth in AI research in general have been reported in the last three decades and especially after 2016 and China and US are the country with the most speed in this regards (17). In terms of countries with highest AI publication in the field of AI and liver cancer, it should be noted that China and USA are leading countries in terms of scientific publications and also have a high number of researchers per million inhabitants, compared to other countries (18, 19). AI with different methods can obtain data from various known and unknown sources and provide relationships between them, in some cases beyond human ability, and also provide diagnostic and therapeutic tools for underserved groups and save time and energy. Therefore, a considerable progress in AI application has been seen and a higher number of related RCTs and prospective studies representing this matter (20). Similar to our results, a recent study evaluated all clinical trials involving AI from 2018 to August 2023 and found that the majority of trials were conducted in the US and China. It is noteworthy that the most common field was gastroenterology among 84 identified trials (21).

Importantly, the plurality of identified articles centered on the analysis of medical images, with a specific emphasis on diagnostic uses. This aligns with a previous work on liver cancer (16) and also other medical fields, where a primary focus has been on interpreting images for disease diagnosis. It’s foreseeable that there will be an increasing number of applications for image-based computer-aided diagnosis systems in the future (3). Our results have demonstrated that approximately 8% of the articles and reviews are related to radiomics. This field involves extracting imaging data from CT, MRI, and PET scans and transforming them into higher-dimensional data. Radiomics is not only used for image diagnosis, but it also integrates data with other patient characteristics to address various aspects of patient care, prognosis, and treatment response prediction (22). A recently published systematic review, evaluating 54 studies, has demonstrated commendable discriminative performance for radiomic features in distinguishing HCC from other solid tumors, predicting microvascular invasion, and forecasting prognosis following locoregional or systemic therapies. Despite the reported overall low quality of the studies, these findings underscore the potential of radiomics in enhancing clinical decision-making for HCC management (23). However, like other AI applications, before radiomics can be clinically implemented, several challenges must be addressed, such as issues with reproducibility, the absence of standardized methodologies, the potential for model overfitting, and the need for robust validation through prospective study designs.

It has been demonstrated that research papers focused on AI-based tumor pathology have exhibited continuous growth since 1999, with the US and China contributing the most to this field respectively (10). Previous reports have demonstrated the utilization of deep neural networks in histopathological diagnosis of various cancers, including prostate and breast cancer (24, 25). Our project revealed that about 6% of publications in the field of AI and liver cancer are associated with histopathological diagnosis and classification.

Genomic evaluation and analysis have proven useful in determining the status of various conditions and diseases, including clinical outcomes and recurrence in breast (26), and ovarian cancers (27), and for classifying viral and bacterial infections (28). We identified instances of utilizing data other than genomics, for treatment response and mortality prediction. Machine learning has utilized data from medical notes (29) and Medicare claims (30) to successfully predict survival in cancer patients beyond 120 days after palliative chemotherapy and short-term mortality in elderly individuals, respectively. In our project specifically related to liver cancer, after medical image analysis, AI models for diagnosis and prognostic prediction, genomic or molecular analysis, and evaluation of clinical outcomes were subjects garnering significant attention. On the other hand, the field of drug discovery exhibited the lowest number of publications.

For these scientometrics projects, we are limited to using the database’s own tools for performing analysis and are not able to merge the search results of different databases together and bring them into one database. Therefore, we were limited to using only one database, which was the reason that we selected Scopus as one of the largest abstract and citation databases and developed a sensitive search strategy for that. However, using the Scopus database for citation counting might have led to an underestimation or overestimation of citations compared to other citation databases. Although there was no language limitation in the search strategy, some publications in a language other than English may not have been indexed in Scopus. In this project, we also found that the majority of the top contributing authors and institutions are from a handful of countries, which may lead to another limitation of our study affecting the generalizability of the findings to a global context.

In this study, we categorized publications related to AI and liver cancer based on their subjects. AI can extract imaging and non-imaging data from different sources and be helpful in the management of patients with cancer in different steps. In preclinical settings, it can guide to drug development or testing new treatment approaches. In cancer imaging studies in radiology and pathology, it can be used with the purposes of classification, detection, segmentation, characterization and monitoring. In the research for cancer therapeutics, it can be used with the purpose of prediction of response to medications, and related complications, prognosis, and survival. Additionally, by determining subgroups of patients with most response to medication with lower rate of complications, it can also be helpful in personalized cancer treatment (31). Still, more studies with collaboration between AI experts, clinicians, and regulatory agencies are urgently needed with a focus on evaluation of external validation of AI applications, assessing their complete impact in clinic settings, using non-imaging input sources from electronic health records like text, number, molecular information, etc., setting up Human-AI collaboration scenarios, and reducing the risk of biases due to training AI applications on datasets with underrepresented groups (20, 32).

More comprehensive work with the inclusion of other databases can be considered in future projects in which they can also explore the connection between the incidence and mortality rates of liver cancer and the quantity of associated research papers in each nation. Co-authorship networks of hyper-prolific authors in this field can be evaluated too. As shown in our project, the number of AI-related publications in liver cancer has had a notable increase from 2019. As some previous works tried to evaluate the effect of the COVID-19 pandemic on publication trends especially about AI (32–34), evaluating how the pandemic affected the publication trends of AI in liver cancer could be an interesting topic for future investigation.

Addressing the ethical dilemmas posed by AI and integrating these methodologies into the curriculum of medical students becomes imperative for future progress. Furthermore, giving due attention to the interplay of human intelligence and artificial intelligence in patient care is a factor that demands consideration.

In conclusion, our study reveals the burgeoning significance of AI in liver cancer research. With a nearly 13-fold growth in the last decade, it is increasingly used in various domains, particularly medical imaging analysis, with significant contributions from China and the US. Our findings underscore the global momentum behind AI-driven advancements. As AI’s role in medical science intensifies, our research highlights its pivotal impact on diagnostics and treatment strategies for liver cancer, ultimately advancing patient care and outcomes.

## Data availability statement

All data analyzed in this project were obtained from Scopus database and can be obtained via this database.

## Author contributions

MR: Conceptualization, Data curation, Formal Analysis, Investigation, Methodology, Writing – original draft. NK: Investigation, Methodology, Writing – review & editing, Data curation. YY: Data curation, Investigation, Methodology, Writing – review & editing, Conceptualization. HK: Data curation, Investigation, Methodology, Writing – review & editing. JL: Conceptualization, Data curation, Investigation, Methodology, Writing – review & editing. CS: Conceptualization, Data curation, Investigation, Methodology, Writing – review & editing. BT: Conceptualization, Data curation, Investigation, Methodology, Writing – review & editing. RS: Data curation, Investigation, Methodology, Writing – review & editing. AW: Data curation, Investigation, Methodology, Writing – review & editing. MN: Data curation, Investigation, Methodology, Writing – review & editing. ZW: Data curation, Investigation, Methodology, Writing – review & editing. JM: Data curation, Investigation, Methodology, Writing – review & editing. DL: Data curation, Investigation, Methodology, Writing – review & editing. AS: Conceptualization, Data curation, Investigation, Methodology, Writing – review & editing. JY: Conceptualization, Data curation, Investigation, Methodology, Project administration, Writing – review & editing.

## Supplementary material

Supplementary Figure 1 Number of Artificial Intelligence-Related Publications on Liver Cancer Based on Publication Types in the Scopus Database by 3rd August 2023.

Supplementary Figure 2 Number and Annual Percentage of Top 10% of Artificial Intelligence-Related Publications (Articles and Reviews) on Liver Cancer by Citation Based on the Scopus Database by September 21^st^ 2023; (1968-2022).

Supplementary Figure 3 Number of Artificial Intelligence-Related Publications (Articles and Reviews) on Liver Cancer Based on the Scopus Database by 3^rd^ August 2023: Top 10 Affiliations.

Supplementary Table 1 The Top 15 Prolific Authors in the Field of Artificial Intelligence and Liver Cancer Based on the Scopus Database by 3^rd^ August 2023.

## Appendix 1. Search Strategy

(TITLE-ABS-KEY (“Hepatocellular* carcinoma*” OR “Liver* Neoplasm*” OR “Liver* Cancer*” OR Hepatoma* OR “Liver Cell Carcinoma*” OR “Cancer of the Liver*” OR “Liver* tumor*” OR “Liver* malignan*”) OR TITLE-ABS-KEY (Hepatocellular* PRE/1 carcinoma*) OR TITLE-ABS-KEY (Liver* OR Hepatic* OR “Hepatic Cell*” PRE/1 Neoplasm* OR Cancer* OR Tumor*)) AND (TITLE-ABS-KEY (“artificial* intelligence*” OR “deep* learning*” OR “machine* learning*” OR “neural* network*” OR “Program* Language*”) OR TITLE-ABS-KEY(“Computer-assisted” OR “Computer-aided” PRE/1 “detection*” OR “diagnosis*”) OR TITLE-ABS-KEY (artificial* PRE/1 intelligence*) OR TITLE-ABS-KEY (deep* PRE/1 learning*) OR TITLE-ABS-KEY (machine* PRE/1 learning*) OR TITLE-ABS-KEY (Program* PRE/1 Language*)).
