# Supplementary material for: Artificial intelligence in liver cancer research: a scientometrics analysis of trends and topics
Source: Front Oncol. 2024 Feb 28;14:1355454. doi: 10.3389/fonc.2024.1355454 (PMC10933055; doi:10.3389/fonc.2024.1355454)
Supplement: Supplementary Figure 1 — Number of Artificial Intelligence-Related Publications on Liver Cancer Based on Publication Types in the Scopus Database by 3rd August 2023. [file Image_1.pdf]

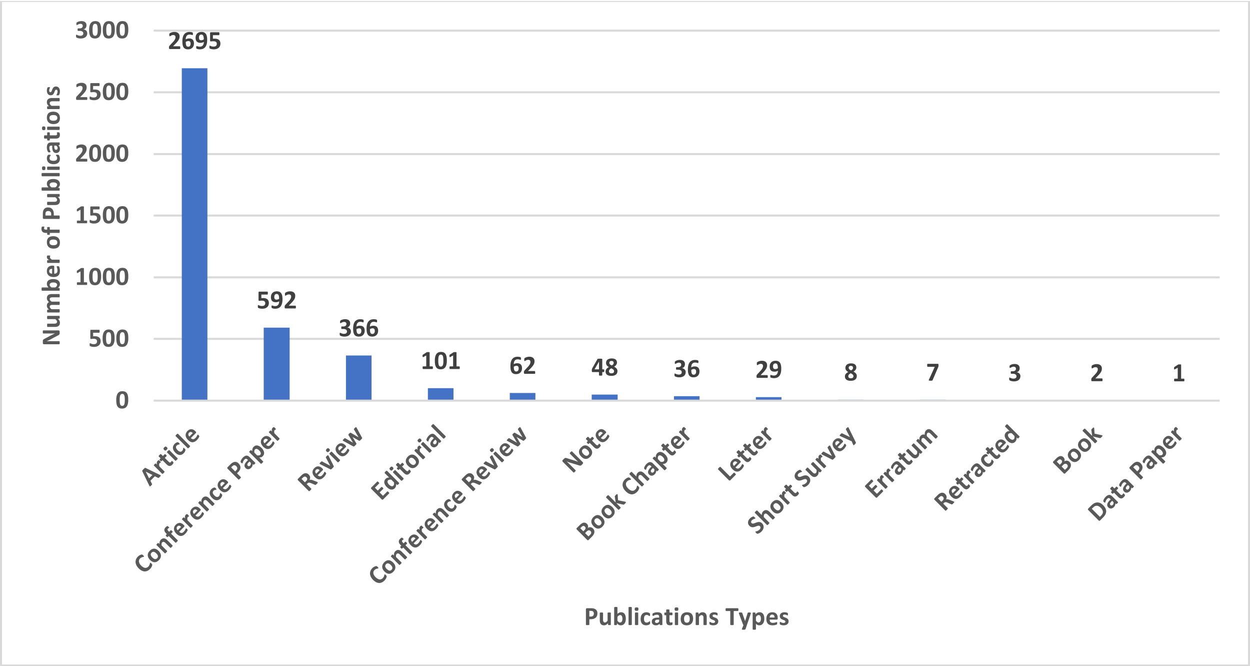

Supplementary Figure 1. Number of Artificial Intelligence-Related Publications on Liver Cancer Based on Publication Types in the Scopus Database by 3rd August 2023

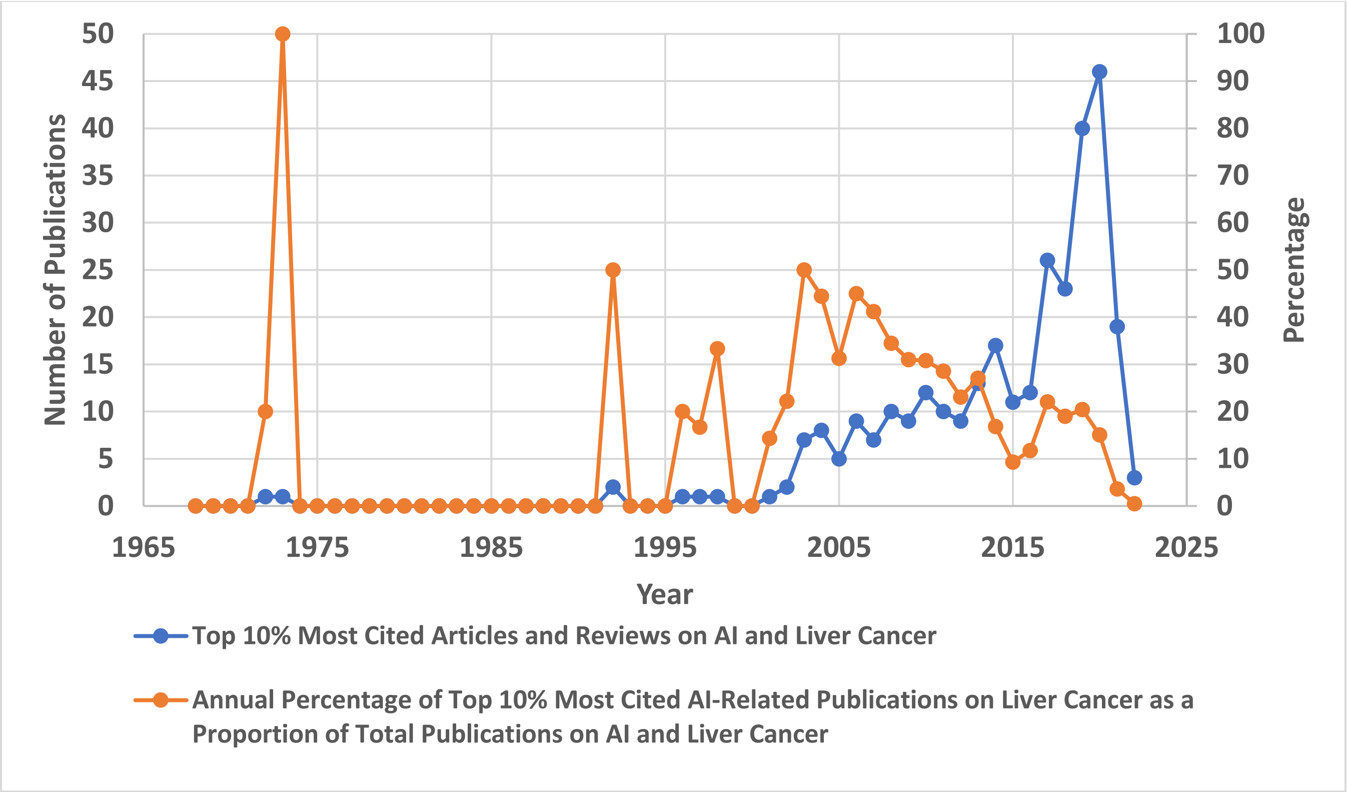

Supplementary Figure 2. Number and Annual Percentage of Top 10% of Artificial Intelligence-Related Publications (Articles and Reviews) on Liver Cancer by Citation Based on the Scopus Database by September 21st 2023; (1968-2022)

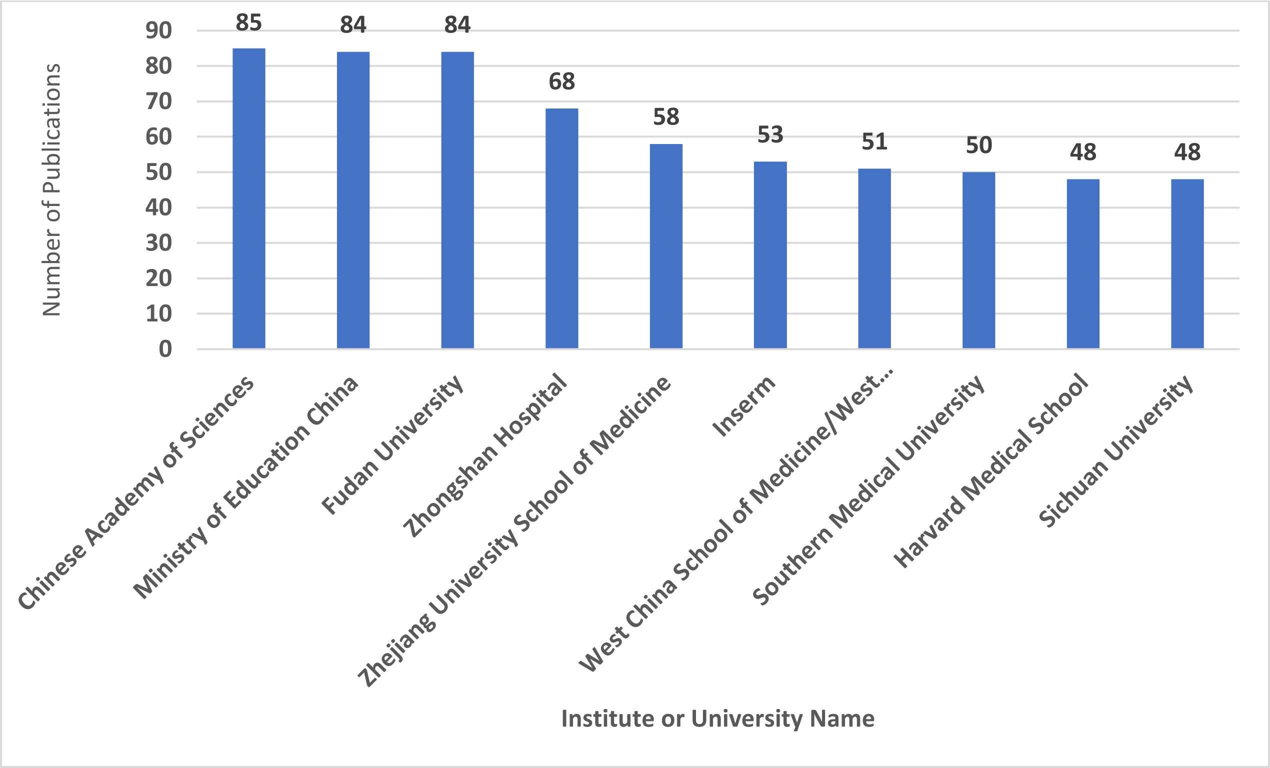

Supplementary Figure 3. Number of Artificial Intelligence-Related Publications (Articles and Reviews) on Liver Cancer Based on the Scopus Database by 3rd August 2023: Top 10 Affiliations
