# Supplementary material for: Artificial intelligence in liver cancer research: a scientometrics analysis of trends and topics
Source: Front Oncol. 2024 Feb 28;14:1355454. doi: 10.3389/fonc.2024.1355454 (PMC10933055; doi:10.3389/fonc.2024.1355454)
Supplement: Supplementary Table 1 — The Top 15 Prolific Authors in the Field of Artificial Intelligence and Liver Cancer Based on the Scopus Database by 3rd August 2023. [file Table_1.docx]

Supplementary Table 1. The Top 15 Prolific Authors in the Field of Artificial Intelligence and Liver Cancer Based on the Scopus Database by 3^rd^ August 2023

| Name | Affiliation | AI Publications | Total Publications | H-Index |
| --- | --- | --- | --- | --- |
| Lee, Jeong Min | Department of Radiology, Seoul National University Hospital, Seoul, South Korea | 24 | 608 | 75 |
| Chapiro, Julius | Yale School of Medicine, New Haven, USA | 16 | 139 | 22 |
| Jiang, Huiyan | Northeastern University, Shenyang, China | 15 | 185 | 18 |
| Tian, Jie | Chinese Academy of Sciences, Beijing, China | 15 | 1297 | 91 |
| Yoon, Jeong-hee | Seoul National University Hospital, Seoul, South Korea | 15 | 170 | 42 |
| Kuang, Ming | Sun Yat-Sen University, Guangzhou, China | 13 | 249 | 41 |
| Sirlin, Claude B | Department of Radiology, San Diego, USA | 13 | 443 | 103 |
| Taouli, Bachir A | Icahn School of Medicine at Mount Sinai, New York, USA | 13 | 250 | 61 |
| Han, Joonkoo | Seoul National University College of Medicine, Seoul, South Korea | 12 | 677 | 77 |
| Joo, Ijin | Seoul National University College of Medicine, Seoul, South Korea | 12 | 161 | 35 |
| Pawlak, Timothy M | The Ohio State University College of Medicine, Columbus, USA | 12 | 1071 | 113 |
| Dietrich, Christoph Frank | Kliniken Hirslanden Beau Site, Bern, Switzerland | 11 | 676 | 76 |
| Marin, Daniele M | Duke University Medical Center, Durham, USA | 11 | 226 | 42 |
| Vilgrain, Valérie | Département of Radiologie, Beaujon Hospital, Université de Paris, Clichy, France | 11 | 609 | 87 |
| Wei, Jingwei | Institute of Automation Chinese Academy of Sciences, Beijing, China | 11 | 44 | 22 |
